# Novel Magnetic Resonance Imaging Findings of Bilaterally Symmetrical Lesions in a Dog With Concomitant Granulomatous Meningoencephalitis and Necrotizing Encephalitis

**DOI:** 10.1111/vru.70117

**Published:** 2025-12-15

**Authors:** Tessa Victoria Procter, Jacqueline Poldy, Adrian William Philbey, Sumari Dancer, Callum Nicholas Atkins, Megan Elizabeth Madden

**Affiliations:** ^1^ The Royal (Dick) School of Veterinary Studies The University of Edinburgh Edinburgh UK

**Keywords:** basal nuclei, canine, caudate nuclei, meningoencephalitis of unknown etiology, meningoencephalitis of unknown origin

## Abstract

A 3‐year‐old, female neutered Bichon Frise was presented with a 2‐day history of behavioral changes and ataxia. The neurological examination was consistent with diffuse forebrain involvement, with left lateralization. An MRI study identified bilaterally symmetrical T2W/T2 fluid‐attenuated inversion recovery (T2‐FLAIR) hyperintense, T1W hypointense, non‐enhancing lesions involving the basal nuclei, with restricted diffusion and hemorrhage affecting both caudate nuclei. Similar bilaterally symmetrical T2W/T2‐FLAIR hyperintense lesions were noted in the hypothalamus, midbrain, and occipital lobes. Histopathological examination indicated concomitant granulomatous meningoencephalitis and necrotizing encephalitis. This case report documents previously unreported bilaterally symmetrical canine brain lesions suspected to be secondary to an autoimmune inflammatory etiology.

**Abbreviations**
AE: autoimmune basal ganglia encephalitis
BPM: beats per minute
CRP: C‐reactive protein
CSF: cerebrospinal fluid
DWI: diffusion weighted imaging
ECG: electrocardiogram
GME: granulomatous meningoencephalitis
MUO: meningoencephalitis of unknown origin
NE: necrotizing encephalitis
NLE: necrotizing leukoencephalitis
NME: necrotizing meningoencephalitis
PCR: polymerase chain reaction
RI: reference interval
SWI: susceptibility weighted imaging
T1W: T1‐weighted
T2‐FLAIR: T2 fluid‐attenuated inversion recovery
T2W: T2‐weighted turbo spin echo
TBEV: tick‐borne encephalitis virus
TE: time to echo
TI: time to inversion
TR: time to repetition

## 1 Signalment, History, and Clinical Findings

A 3‐year‐old, 6.9 kg, female neutered Bichon Frise dog was referred to the Neurology and Neurosurgery Department at the University of Edinburgh with a 2‐day history of acute onset behavioral changes, including inappropriate vocalization, aggression, and ataxia. The dog had no travel history or prior medical conditions and was up to date with preventative vaccinations, flea, and worm treatments.

On physical examination, the patient was markedly obtunded, with paroxysmal episodes of aggression and vocalization of short duration (several seconds). Bradycardia (48 BPM) with second‐degree atrioventricular block was noted on electrocardiogram (ECG). Serial blood pressure measurements were within normal limits. Neurological examination, performed by an ECVN‐certified neurologist (MM), revealed marked obtundation and inappropriate behavior with extreme vocalization during manipulation, cervical ventroflexion with intermittent left‐sided pleurosthotonus, nonambulatory tetraparesis with postural reaction deficits in all limbs (right limbs more severely affected), inconsistent menace response bilaterally (right eye more severely affected), and reduced oculocephalic reflexes (right eye more severely affected) (Video 1). The remainder of the neurological examination was normal. These findings were consistent with diffuse forebrain neurolocalization, with left lateralization.

**VIDEO 1:** Neurological examination demonstrating obtundation and inappropriate behavior with episodes of extreme vocalization, cervical ventroflexion with intermittent left‐sided pleurosthotonus, and nonambulatory tetraparesis with postural reaction deficits in all four limbs (right limbs more severely affected).

Given the presentation, signalment, and examination findings, differential diagnoses included inflammatory non‐infectious disease (meningoencephalitis of unknown origin/etiology [MUO]), inflammatory infectious disease (viral, bacterial, protozoal, or fungal meningoencephalitis), and neoplastic disease (primary or metastatic), with metabolic/toxic causes considered less likely given the subtle lateralization of neurological signs.

## 2 Imaging, Diagnosis, and Outcome

Complete blood count and biochemistry revealed mild thrombocytopenia (94 × 10^9^/L, reference interval [RI]: 200–500 × 10^9^/L), markedly elevated C‐reactive protein (CRP) (64.1 mg/L, RI: 0.0–5.0 mg/L), elevated creatine kinase (692 U/L, RI: 50–200 U/L), and low triglycerides (0.33 mmol/L, RI: 0.57–1.14 mmol/L). Urinalysis (free catch) showed a specific gravity of 1.051 and borderline proteinuria (0.23, RI: <0.2).

Echocardiography, performed to rule out cardiac causes given the bradycardia and second‐degree atrioventricular block, revealed no structural abnormalities. Bradycardia was presumed secondary to high vagal tone, given the conversion to sinus rhythm on stimulation. This may be mediated by pathology involving autonomic nuclei within the medulla oblongata or an early sign of raised intracranial pressure [1].

Magnetic resonance imaging was performed under general anesthesia using a 1.5 Tesla magnet (Avanto, Siemens Healthineers, Erlangen, Germany) and a 15‐channel knee coil. Sequences included sagittal and dorsal T2W (time to repetition [TR]: 4000 ms, time to echo [TE]: 90 ms), transverse T2W (TR: 4620 ms, TE: 90 ms), T1W (TR: 557 ms, TE: 11 ms) pre‐ and 2 min post‐intravenous contrast administration (0.1 mmol/kg gadoteric acid, Dotarem, Guerbet, Villepinte, France), T2* gradient‐echo (GRE) (TR: 800 ms, TE: 20 ms, flip angle: 20°), susceptibility weighted imaging (SWI) (TR: 49 ms, TE: 40 ms, flip angle: 15°), T2 fluid‐attenuated inversion recovery (T2‐FLAIR) (TR: 8000 ms, TE: 123 ms, time to inversion [TI]: 2400 ms), and diffusion weighted imaging (DWI) (TR: 7100 ms, TE: 134 ms). Slice thickness was 2.5 mm, except for the SWI (1.6 mm), and interslice spacing was 2.75 mm.

**FIGURE 1:**
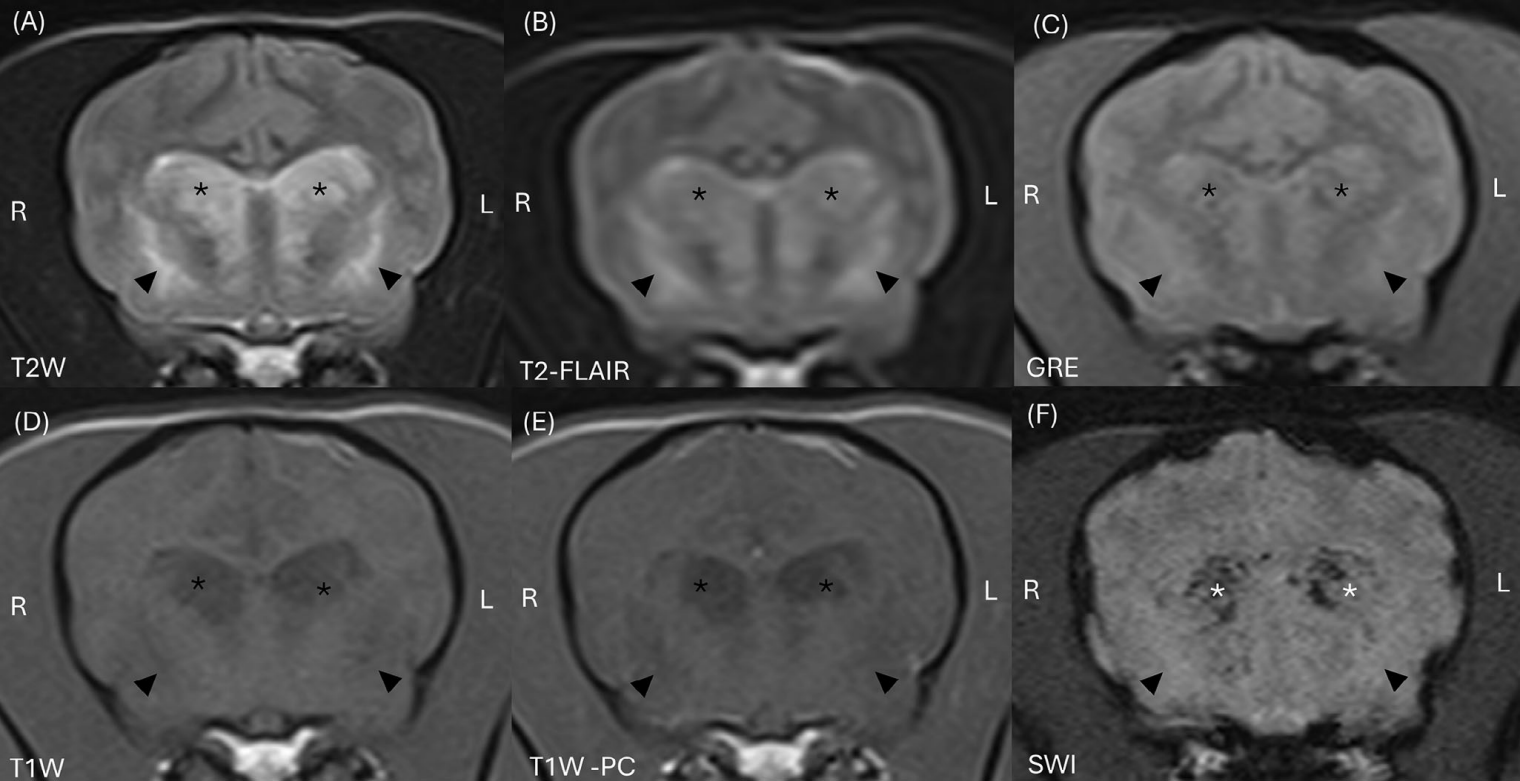
MRI of the head of a dog at the level of the caudate nuclei: (A) transverse T2W (TR 4620 ms, TE 90 ms, slice thickness 2.5 mm, slice interval 2.75 mm), (B) transverse T2‐FLAIR (TR 800 ms, TE 123 ms, slice thickness 2.5 mm, slice interval 2.75 mm), (C) transverse GRE (TR 800 ms, TE 20 ms, slice thickness 2.5 mm, slice interval 2.75 mm), (D) transverse T1W (TR 557 ms, TE 11 ms, slice thickness 2.5 mm, slice interval 2.75 mm), (E) transverse T1W post‐contrast (TR 557 ms, TE 11 ms, slice thickness 2.5 mm, slice interval 2.75 mm), (F) transverse SWI (TR 49 ms, TE 40 ms, slice thickness 1.6 mm, slice interval 2.75 mm). Bilaterally symmetrical T2W (A) and T2‐FLAIR (B) hyperintense, T1W hypointense (C) with no contrast enhancement (T1W post‐contrast, (D) lesions within the caudate nuclei (asterisk, *) and lentiform nuclei (arrowheads). Right is to the left of the images. GRE, gradient‐echo; SWI, susceptibility weighted imaging; T2‐FLAIR, T2 fluid‐attenuated inversion recovery.

**FIGURE 2:**
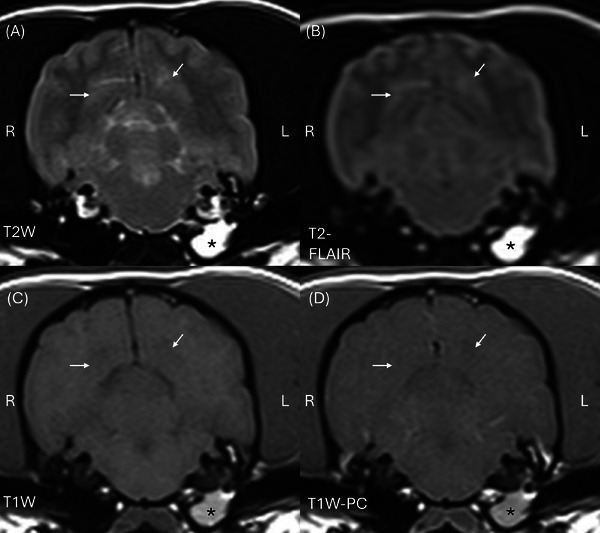
MRI of the head of a dog at the level of the occipital lobes: (A) transverse T2W (TR 4620 ms, TE 90 ms, slice thickness 2.5 mm, slice interval 2.75 mm), (B) transverse T2‐FLAIR (TR 800 ms, TE 123 ms, slice thickness 2.5 mm, slice interval 2.75 mm), (C) transverse T1W (TR 557 ms, TE 11 ms, slice thickness 2.5 mm, slice interval 2.75 mm), (D) transverse T1W post‐contrast (TR 557 ms, TE 11 ms, slice thickness 2.5 mm, slice interval 2.75 mm). Bilaterally, relatively symmetrical T2W (A) and T2‐FLAIR (B) hyperintense, T1W hypo to isointense (C) with no contrast enhancement (D) lesions within the occipital lobes (arrows). There is T2W, T2‐FLAIR, and T1W hyperintense, non‐enhancing material in the left tympanic bulla (asterisk, *). Right is to the left of the images. T2‐FLAIR, T2 fluid‐attenuated inversion recovery.

The MRI study (interpreted by a second‐year ECVDI resident (TP) under ECVDI‐certified radiologist supervision (SD)) indicated bilaterally symmetrical, heterogeneous but predominantly T2W and T2‐FLAIR hyperintense, T1W markedly hypointense (relative to grey and white matter), non‐contrast‐enhancing lesions in the caudate nuclei (Figure 1). The caudate nuclei were markedly enlarged, causing moderate mass effect on the neighboring lateral ventricles. Additionally, the caudate nuclei contained patchy regions of SWI and GRE signal void and demonstrated restricted diffusion on DWI (Figure 1C,F). Bilaterally symmetric T2W and T2‐FLAIR hyperintense, T1W hypo‐isointense to white matter, non‐enhancing lesions were also observed in the region of the lentiform nuclei and claustrum. Similar, less well‐defined lesions were visible within the hypothalamus, extending caudally into the subthalamus and into the ventral aspect of the midbrain. Further, subtle T2W and T2‐FLAIR hyperintense, T1W hypo‐isointense lesions, were noted bilaterally in the cortical grey matter of the occipital lobes (Figure 2). There was no evidence of pachy‐ or leptomeningeal contrast enhancement. Reduced T2W cerebrospinal fluid (CSF) signal within the cerebral sulci was consistent with raised intracranial pressure. In summary, the MRI findings were consistent with a multifocal, predominantly bilaterally symmetrical, polioencephalopathy. Incidentally, a left‐sided otitis media was noted. Additionally, there was caudal indentation of the cerebellum by the occipital bone, suggestive of grade 1 Chiari‐like malformation [2].

Considering the striking bilaterally symmetrical lesion distribution, differential diagnoses were reconsidered, with metabolic/degenerative (e.g., thiamine deficiency [3, 4] and mitochondrial encephalopathy [5, 6, 7, 8, 9]) or toxic causes prioritized. Infectious etiologies with bilaterally symmetrical imaging findings (e.g., tick‐borne encephalitis virus (TBEV) [10, 11, 12] or fungal meningoencephalitis [13, 14]) remained included. Following review of the human literature, a vascular cause (e.g., deep cerebral vein thrombosis [15]) was also considered. Autoimmune (MUO) and neoplastic (e.g., gliomatosis cerebri and infiltrative lymphoma) causes were considered less likely given the bilaterally symmetrical nature of the findings. The history was reviewed, establishing that the risk of toxin exposure was low.

CSF analysis, performed by an ECVP resident under the supervision of an ACVP‐certified clinical pathologist, demonstrated marked neutrophilic to mixed pleocytosis (total nucleated cell count 2376.0/µL RI: 0–5.0/µL, protein 94.0 mg/dL RI: <25 mg/dL). Nucleated cells comprised 69% non‐degenerate neutrophils, 6% small lymphocytes, and 25% monocytoid cells. No microorganisms were identified. Potential causes for a marked neutrophilic pleocytosis include infectious meningoencephalitis (bacterial or fungal) [16, 17], cerebrovascular accident [18, 19, 20], MUO [17, 20], metabolic (e.g., thiamine deficiency [16, 17, 20, 21]), or neoplasia (e.g., lymphoma [17, 20]). Considering the acute presentation and bilaterally symmetrical MRI findings, neutrophilic invasion of the CSF associated with a severe necrotic process secondary to an acute toxic/metabolic insult was also considered. Subsequent bacterial and yeast cultures on CSF were negative.

Further tests were undertaken to investigate infectious, metabolic, and systemic neoplastic or inflammatory disease. Antibodies for *Anaplasma phagocytophilum*, *Anaplasma platys*, *Borrelia burgdorferi*, *Ehrlichia canis*, *Ehrlichia wingii*, *Ehrlichia chaffeensis*, and *Dirofilaria immitis* were all negative (Idexx SNAP4Dx, Idexx Laboratories, Wetherby, UK). Serologies for *Toxoplasma gondii* and *Neospora caninum* were negative. Serologies and CSF polymerase chain reaction (PCR) for TBEV were negative. The blood thiamine concentration was within normal limits (70 µg/L, RI 46–112 µg/L). The blood cobalamin concentration was mildly decreased (233 ng/L, RI: >275 ng/L). The serum thyroid‐stimulating hormone activity was within reference (0.045 ng/mL, RI: 0.00–0.50 ng/mL), and total thyroxine was decreased (<13.0 nmol/L, RI: 15.00–48.00 nmol/L), consistent with euthyroid sick syndrome. Coagulation tests (prothrombin time and activated partial thromboplastin time) and viscoelastic monitoring were within normal limits. Thoracic radiographs revealed no abnormalities, and abdominal ultrasonography findings were non‐specific, with bilateral nephropathy (considered chronic degenerative changes), mild hepatopathy (most likely benign, e.g., vacuolar changes), mild splenomegaly (likely sedation‐related), and incidental gallbladder precipitates.

Pending the aforementioned diagnostic test results, treatment was initiated with thiamine and cobalamin supplementation for metabolic causes, analgesia (methadone (Synthadon, AnimalCare Ltd, York, UK), paracetamol (Paracetamol, B. Braun, Melsungen, Germany), gabapentin (Gabapentin, Tillomed Laboratories Ltd, Luton, UK)), levetiracetam (Desitrend, Destin Pharma Ltd, Milton Keynes, UK), and supportive care (maropitant (Prevomax, Dechra Veterinary Products, Hadnall, UK) and intravenous fluid therapy). After 4 days of hospitalization, the patient remained static neurologically, but an increased frequency of aggressive and vocalization episodes was observed. Treatment with an anti‐inflammatory dose (0.15 mg/kg intravenous) of dexamethasone (Colvasone, Norbrook, Market Harborough, UK) was initiated. The patient's neurological status significantly improved in response to corticosteroid treatment, becoming ambulatory within 24 h, with a reduction in the frequency of spontaneous vocalization episodes. However, the patient continued to demonstrate extreme aggression when handled. Despite the profound neurological improvement, the owners elected euthanasia due to concerns regarding the persistent aggression.

**FIGURE 3:**
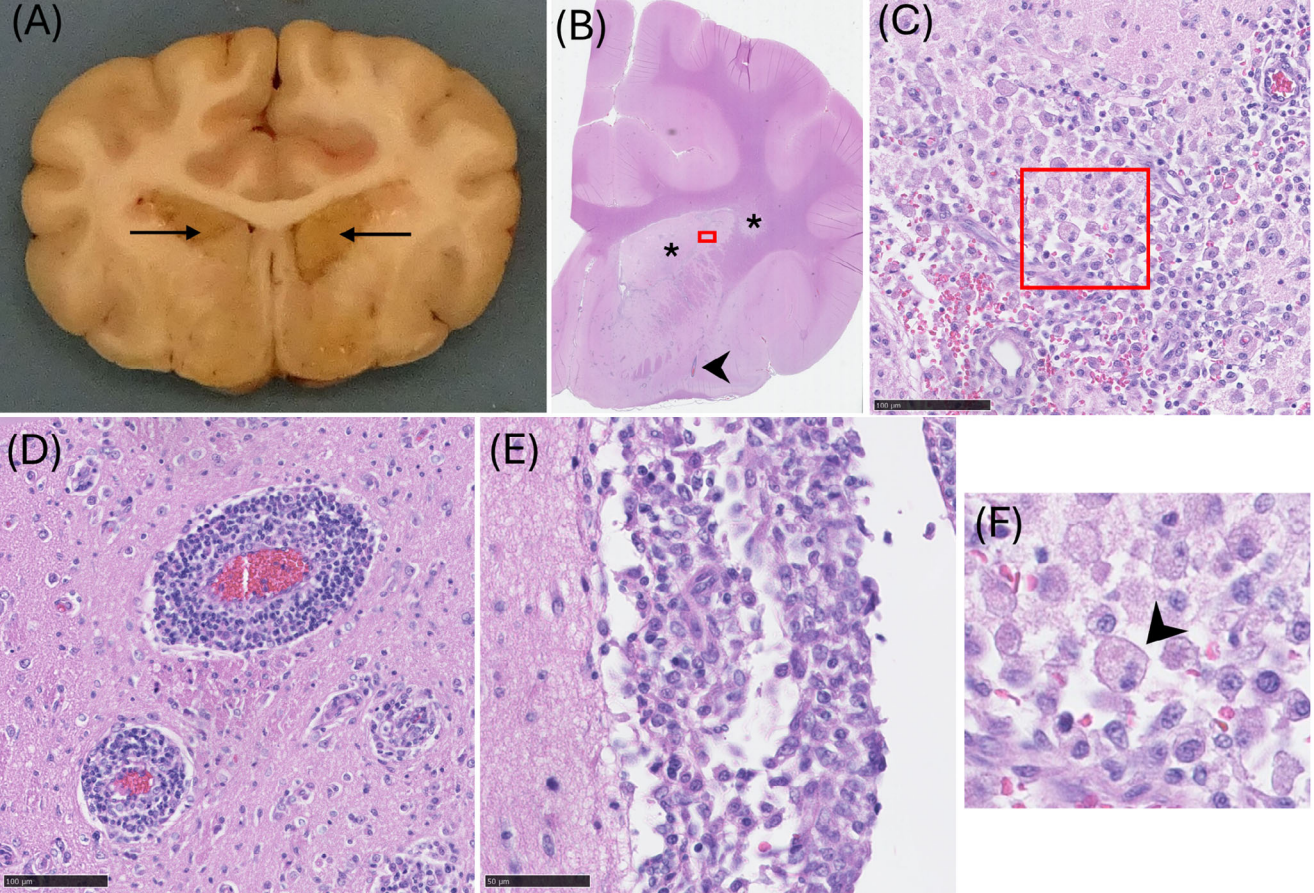
Histological lesions of granulomatous and necrotizing meningoencephalitis. (A) Gross lesions of granulomatous meningoencephalitis, showing bilaterally symmetrical tan‐brown areas of necrosis predominantly affecting the caudate nuclei (arrows). Formalin‐fixed tissue. (B) There is rarefaction of the caudate nucleus and adjacent white matter (asterisks), and prominent perivascular cellular infiltrates (arrowhead). The red box indicates the area from which (C) was acquired. (C) Malacia within the caudate nucleus, characterized by numerous gitter cells, a zoomed version of the gitter cell (arrowhead) is visible in (F). HE. Scale bar = 100 µm. Hematoxylin and eosin (HE). (D) Perivascular cuffs are composed of dense lymphocytic infiltrates. HE. Scale bar = 100 µm. (E) Lymphohistiocytic infiltrates are present within the meninges. HE. Scale bar = 50 µm.

A comprehensive postmortem examination was performed by an ECVP second‐year resident (JP) under FRCPath‐certified pathologist supervision (AP), which was macroscopically unremarkable. Following fixation (48 h, neutral buffered 10% formalin), transverse sectioning of the brain revealed bilaterally symmetrical, well‐demarcated tan discoloration of the caudate nuclei (Figure 3A). Histologically, these areas were characterized by marked, locally extensive encephalomalacia, with infiltration by innumerable Gitter cells. Dense lymphocytic to lymphohistiocytic perivascular cuffs, including some epithelioid cells, were distributed throughout the brain (Figure 3C–F). Multifocal to locally extensive perivascular infiltrates were most severe in the grey and white matter of the frontal lobes, the basal nuclei, and the internal capsule. Lymphohistiocytic perivascular cuffing was also present, without necrotic foci, in the midbrain and pons. A locally extensive, histiocytic leptomeningitis was also present at the ventral aspect of the frontal lobes. Histology confirmed a diagnosis of granulomatous meningoencephalitis (GME) with concurrent necrotizing encephalitis (NE).

## 3 Discussion

Here, we describe novel MRI findings of multifocal, bilaterally symmetrical intraparenchymal brain lesions in a dog with a histopathological diagnosis of concomitant GME and NE. Upon initial presentation, an inflammatory etiology was considered most likely given the patient's signalment and neurological examination findings [22, 23, 24, 25]. However, in view of the unusual MRI findings of bilaterally symmetrical lesions, predominantly involving the basal nuclei, and sequence characteristics consistent with severe necrosis of the caudate nuclei, an inflammatory etiology was deprioritized, resulting in a delay to initiate appropriate treatment. This case emphasizes the importance of integrating presentation, signalment, and neurological examination findings when interpreting MRI studies and highlights the potential for autoimmune inflammatory etiologies to produce bilaterally symmetrical lesions.

Typically, in small animals, bilaterally symmetric MRI lesions are associated with metabolic and degenerative disorders [26]. One of the most well‐recognized metabolic conditions is thiamine deficiency, with bilaterally symmetric, T2W and T2‐FLAIR hyperintense, variably enhancing lesions most commonly in the red, lateral geniculate, and vestibular nuclei, as well as the caudal colliculi [3, 27, 28, 29]. However, bilaterally symmetric, T2W and T2‐FLAIR caudate nuclei hyperintensities with mass effect have been reported in a 3‐year‐old Golden Retriever with an acute onset of behavioral changes and confirmed severe thiamine deficiency [4]. In humans, Wernicke's encephalopathy, a neurological syndrome caused by thiamine deficiency, shows bilaterally symmetric T2W/T2‐FLAIR hyperintensities in multiple regions [30, 31, 32], with caudate nuclei affected in more atypical forms [33]. Therefore, given the MRI findings mirroring those reported in both canines and humans, thiamine deficiency was considered a possible differential diagnosis; however, it was subsequently ruled out on the basis of our patient's normal whole‐blood values.

Toxic causes, including carbon monoxide toxicity, are also known to yield bilaterally symmetrical lesions involving the basal nuclei on MRI studies [26, 34] but were considered less likely in our patient, given no suggestive history. Similarly, infectious causes, including TBEV and coccidiomycosis, which show similar MRI characteristics to our patient, with bilaterally symmetrical grey matter lesions affecting the basal nuclei [10, 11, 12, 13, 14], were ruled out with further diagnostic tests and histopathology.

Bilaterally symmetrical brain lesions involving the caudate nuclei have also been reported on MRI studies of dogs with confirmed neurodegenerative disorders, that is, metabolic encephalopathy secondary to mitochondrial dysfunction [5] and degenerative encephalopathy in the Nova Scotia duck tolling retriever [35]. However, in contrast to the case we describe, lesions are typically T2W hyperintense, T2‐FLAIR and T1W hypointense, and non‐contrast‐enhancing, consistent with chronic cavitary lesions confirmed on postmortem examination. Dogs with neurodegenerative disorders typically present at a young age and display a chronic, progressive disease pattern. However, acute presentations have been described. Human subacute necrotizing encephalomyelopathy (Leigh's syndrome) secondary to mitochondrial dysfunction is characterized by symmetrical brainstem and basal nuclei lesions [36, 37]. Canine forms of subacute necrotizing encephalomyelopathy have been reported with similar MRI findings to the patient we describe; however, the caudal colliculi, vestibular, and cerebellar nuclei are also affected. Areas of cavitation and encephalomalacia were confirmed on postmortem examination [9, 38, 39, 40, 41, 42]. In summary, neurodegenerative disorders can present acutely with bilaterally symmetrical lesions involving the caudate nuclei, similar to those described in our patient, supporting our consideration of this etiology.

In dogs, bilaterally symmetric brain lesions are also reported with vascular causes such as hypertensive encephalopathy. However, these lesions typically display a predilection for the white matter tracts [43, 44, 45]. In humans, deep cerebral venous thrombosis can result in bilateral thalamic lesions and can affect the basal nuclei and corpus callosum [46]. Lesions are T2W hyperintense with evidence of restricted diffusion and GRE signal voids [46, 47], reflecting the imaging findings observed in our patient. However, asymmetric lesions have also been shown [48]. Cerebral venous thromboses are rarely reported in dogs, with one case report showing multifocal, asymmetric postmortem changes, including edema, hemorrhage, and necrosis affecting the basal nuclei and thalamus [49]. CSF neutrophilic pleocytosis, more commonly associated with inflammatory conditions, has been reported with canine ischemic myelopathies [50] and cerebrovascular accidents [18, 20, 21]. Given the acute presentation of our patient, in combination with the MRI and CSF findings, a vascular cause was considered, even though imaging findings for deep cerebral venous thrombosis have not been reported in dogs.

In dogs, suspected autoimmune inflammatory conditions such as MUO commonly cause focal, multifocal, or diffuse asymmetric lesions with varying degrees of white/grey matter involvement, intraparenchymal contrast enhancement, meningeal contrast enhancement, and mass effect, depending on the subtype [51], for example, GME or NE, the latter subdivided into necrotizing meningoencephalitis (NME) and necrotizing leukoencephalitis (NLE) depending on lesion predilection for the grey or white matter, respectively [25, 52, 53]. Initially, an inflammatory etiology was prioritized in our case, due to the typical signalment (young to middle‐aged, small breed, female [51]) and neurological examination findings. GME and NE can present similarly, with overlapping MRI features [53, 54]. For GME, typical MRI findings include asymmetric multifocal grey and white matter or focal lesions with irregular margins and variable mass effect. The lesions are T2W and T2‐FLAIR hyperintense, with variable T1W intensity changes. Additionally, the lesions are variably contrast‐enhancing, and there is rarely meningeal enhancement [24, 25, 54]. These MRI findings differ from those in our patient in that the lesions were symmetrically distributed, with no contrast or meningeal enhancement. In NE, lesions are more commonly localized to the forebrain, although brainstem involvement has been reported in NLE [24, 25, 51]. However, like GME, NE lesions are commonly asymmetrically distributed, with irregular margins, variable contrast enhancement, hyperintense T2W, and variable T1W signal intensity changes [23, 24, 25]. Concomitant GME and NE have been reported in four small breed dogs; however, lesions were asymmetrically distributed with variable degrees of contrast enhancement [55], differing from changes seen in our patient. There is a single report of bilaterally symmetrical MUO in a dog; however, lesions were localized to the cranial nerves [56]. Furthermore, neutrophilic pleocytosis is rarely seen with MUO (<10% cases) [24] making both the CSF and imaging findings in our case atypical for an inflammatory etiology.

Bilaterally symmetric lesions involving the basal nuclei have been reported in an inflammatory autoimmune condition in humans known as autoimmune basal ganglia encephalitis (AE) [57]. Clinical signs vary with seizures, movement disorders, and behavioral changes reported [25, 58, 59, 60]. The limbic subtype is the most common form in humans, where bilateral medial temporal lobe structure signal changes are a diagnostic criterion [57, 60]. However, there are reports of caudate nucleus and putamen changes with similar signal intensity descriptions to our patient [58, 59, 60, 61]. Furthermore, in humans with AE, contrast enhancement is uncommon, and changes are usually bilateral [57]. Although bilaterally symmetric autoimmune inflammatory lesions have not been reported in canine patients, they have been reported in human disease and mirror some, but not all, of the MRI features seen in our patient.

We believe that the persistent behavioral changes and aggression observed in our patient were due to the severe pathology involving the caudate nuclei. Caudate nucleus dysfunction, as seen in Huntington's disease [62, 63] and schizophrenia [64], can lead to aggression and other behavioral changes. This is due to its crucial role in regulating behavior through connections with the prefrontal cortex, which governs impulse control and decision‐making [65]. Involvement of the caudate nuclei should be considered in dogs presenting with aggression as a clinical sign, particularly when associated with other signs of forebrain involvement.

In conclusion, this case is unique in that it reports concomitant GME and NE in a Bichon Frise, a combination not previously documented in this breed [55]. It highlights the importance of considering patient signalment when formulating differential diagnoses and supports the consideration of autoimmune etiologies in cases of bilaterally symmetrical MRI lesions.

## Author Contributions

Conception and design: Megan Elizabeth Madden and Tessa Victoria Procter. Acquisition of data: Megan Elizabeth Madden, Tessa Victoria Procter, Jacqueline Poldy, and Callum Nicholas Atkins. Analysis and Interpretation of data: Megan Elizabeth Madden, Tessa Victoria Procter, Jacqueline Poldy, Sumari Dancer, Adrian William Philbey, and Callum Nicholas Atkins. Drafting the article: Megan Elizabeth Madden, Tessa Victoria Procter, and Jacqueline Poldy. Reviewing article for intellectual content: Megan Elizabeth Madden, Tessa Victoria Procter, Jacqueline Poldy, Sumari Dancer, Adrian William Philbey, and Callum Nicholas Atkins. Final approval of the completed article: Megan Elizabeth Madden, Tessa Victoria Procter, Jacqueline Poldy, Sumari Dancer, Adrian William Philbey, Callum Nicholas Atkins. Agreement to be accountable for all aspects of the work in ensuring that questions related to the accuracy or integrity of any part of the work are appropriately investigated and resolved: Megan Elizabeth Madden, Tessa Victoria Procter, Jacqueline Poldy, Sumari Dancer, Adrian William Philbey, and Callum Nicholas Atkins.

## Disclosure

No previous presentations or publications have been produced associated with this work. A CARE checklist was used for case reports.

## Ethics Statement

The University of Edinburgh ethical guidelines for case reports were followed when undertaking this work. A high standard (best practice) of veterinary care was followed, and informed client consent was given.

## Conflicts of Interest

The authors declare no conflicts of interest.

## Data Availability

Contact the correspondence addressee for further information.
